# Correction: Order Under Uncertainty: Robust Differential Expression Analysis Using Probabilistic Models for Pseudotime Inference

**DOI:** 10.1371/journal.pcbi.1005477

**Published:** 2017-04-11

**Authors:** The PLOS Computational Biology Staff

## Notice of Republication

This article was republished on November 28, 2016, to correct an error in Figure 9 that was introduced during the typesetting process. The publisher apologizes for the error. Please download this article again to view the correct version. The originally published, uncorrected article and the republished, corrected articles are provided here for reference.

## Supporting information

S1 File Originally published, uncorrected article. (PDF)

S2 File Republished, corrected article. (PDF)
