# Supplementary material for: Order Under Uncertainty: Robust Differential Expression Analysis Using Probabilistic Models for Pseudotime Inference
Source: PLoS Comput Biol. 2016 Nov 21;12(11):e1005212. doi: 10.1371/journal.pcbi.1005212 (PMC5117567; doi:10.1371/journal.pcbi.1005212)
Supplement: S1 Text — (PDF) [file pcbi.1005212.s001.pdf]

Supplementary Materials

Kieran Campbell and Christopher Yau

October 31, 2016

Contents

1 Supplementary Text **1**
1.1 Inherent uncertainty in point-estimation methods 1
1.2 Consistency with Monocle 1
1.3 Data Analysis 1
1.4 Differential expression analysis 3

1 Supplementary Text

1.1 Inherent uncertainty in point-estimation methods

To demonstrate that there is inherent uncertainty in pseudotime (i.e. it's not just the consequence of using a probabilistic model) we subsampled cells, recomputed the pseudotime for each subsample and computed the variance across subsamples. In particular, we recomputed the pseudotime estimate using `Monocle`'s MST fitting and the Laplacian Eigenmaps embedding as above for 30 resamples to 80% of the total number of cells (without replacement). For each subsample, the pseudotimes were standardized to lie in $[0, 1)$. Since pseudotimes are equivalent up to a parity transformation, if the correlation between the pseudotimes assigned at each resample and using the entire cell set was less than 0 then the pseudotimes were rescaled to $\tilde{t} = 1 - t$ to 'orient' them in the right direction[1]. Supplementary Figure S2 shows the 2σ interval[2] across all cells. It can be seen to vary to as much as 0.5, implying there is a large inherent uncertainty in pseudotimes even using point-estimate methods.

1.2 Consistency with `Monocle`

To ensure the GPLVM fit is consistent with other methods we compared the MAP pseudotime estimates with those assigned using `Monocle`. `Monocle` works by using Independent Component Analysis (ICA) for dimensionality reduction then fits a minimum spanning tree (MST) in the reduced space. The longest path through the minimum spanning tree is taken to be the trajectory and the pseudotime of each cell is the length along this trajectory.

We performed two comparisons: firstly using the entire `Monocle` method (using the 500 most variable genes for dimensionality reduction), and secondly using just the MST fitting using the Laplacian Eigenmaps embedding as described above. The results can be seen in Supplementary Figures S3 with R^2 values of 0.83 and 0.96. Also plotted are the 95% HPD credible intervals, and in general the `Monocle` value is captured within this interval.

1.3 Data Analysis

All analyses are available either as Rmarkdown notebooks or as `R` scripts at `http://github.com/kieranrcampbell/pseudogp-paper/`

[1] Note that this transformation actually gives benefit of the doubt to each resample and will only bias the variance downwards.

[2] Approximately equivalent to the 95% CI

1.3.1 Trapnell

The data was imported from the `HSMMSingleCell` package[3] and $\log_{10}$ of the FPKM values with a pseudo-count of 1. Then, using genes with a transformed expression greater than 0.3 a generalized linear model was fit with $\text{CV}^2 \sim a 10^{-k\mu}$ where CV^2 is the square of the coefficient of variation and μ is the mean for a particular gene. Genes whose measured coefficient of variation was greater than four times that predicted by the model were then used for dimensionality reduction, with the reasoning that those that vary greatest will contribute most to pseudotemporal processes.

The Laplacian Eigenmaps embedding was then found using the `R` package `embeddr`[4] using the default parameters. The Trapnell dataset contains a differentiation trajectory of differentiating myoblasts as well as contaminating interstitial mesenchymal cells. These mesenchymal cells were discovered using Gaussian Mixture Model clustering in the reduced space (using $k = 3$ components) and subseqently removed. A further four cells were removed as outliers on the manifold. This reduced the original 271 cell dataset to 155 cells. The PCA and t-SNE representations were found using the `R` package `scater`[5] using the default gene set (the 500 most variable genes) and the reduced set of 155 cells as discovered above. For the t-SNE representation a perplexity of 3 was used.

The Bayesian GPLVM pseudotime trajectory was fitted on the Laplacian Eigenmaps embedding using the `R` package `pseudogp`[6] using the default MCMC parameters and smoothing hyperparameters $\gamma_\alpha = 30$, $\gamma_\beta = 5$ which were chosen empirically based on the quality of the fit.

1.3.2 Burns

The data were downloaded from the Gene Expression Omnibus accession number 71982 (`http://www.ncbi.nlm.nih.gov/geo/query/acc.cgi?acc=GSE71982`) as the pre-processed TPM count matrix. The transformation applied to the TPM counts was performed identically to the original paper: genes with TPM expression less than 1 are set to 0 while those with TPM expression greater than 1 are set to $\log_2$ of that expression level. The original publication focussed pseudotime analyses on Utrichular cells rather than Cochlear ones. Using "Ute_P1" in the column names of the cells in the data as designating Utrichular cells, we took only those forward for analysis.

Then a PCA plot (similar to the original paper) was constructed once more using `scater`. The original publication states the "top" 195 genes were used for PCA analysis and we emailed the corresponding author asking the definition of "top", but received no reply so assumed it refers to "most variable". Using this definition we found a similarly-shaped PCA plot to that in the publication. The pseudotime trajectory identified in the paper involves only a subset of cells that represent a supporting cell to hair cell transition. We identified these cells in the PCA by plotting the intensity of the fluorescent markers measured as well as three marker genes (*LFNG*, *CDH4* and *SOX2*) and computationally isolated these using k-means clustering.

The Laplacian Eigenmaps embedding of just the trajectory cells was found using the `embeddr` `R` package with 35 nearest-neighbours and the entire gene-set. Two cells were removed from the analysis as outliers. A principal curve[7] was fitted and the behaviour of several marker genes observed to be identical to that shown in the original publication, implying we had recovered the same cell ordering. For the PCA representation of the differentiation the `scater` defaults were used but with `scale_features = FALSE`. For the t-SNE representation, a range of perplexities were examined to find one that most gave a trajectory like structure in the reduced embedding. A perplexity of 2 was subsequently chosen, with all other parameters those of the defaults in `scater` except `scale_features = FALSE`.

The GPLVM was fitted on the Laplacian Eigenmaps embedding using `pseudogp`. The curve was initialised from a principal curve and smoothing hyperparameters $\gamma_\alpha = 20$, $\gamma_\beta = 2$ were used.

[3] `http://bioconductor.org/packages/release/data/experiment/html/HSMMSingleCell.html`
[4] `http://github.com/kieranrcampbell/embeddr/`
[5] `http://github.com/davismcc/scater`
[6] `http://kieranrcampbell.github.com/pseudogp`
[7] Which can be thought of as representing the MAP of a 1D GPLVM

1.3.3 Shin

The data were downloaded as supplementary data from `http://www.cell.com/cms/attachment/2038326541/2052521610/mmc7.xlsx`. This included the `Waterfall` pseudotime assignment, allowing us to compare as a sanity check. The PCA representation was found using the top 195 most variable genes and `scale_features = FALSE` in the `scater` package. The shape closely resembled that from the original publication and colouring by the pseudotime assigned by `Waterfall` clearly showed this was the case. The t-SNE representation was found again using `scater` with 195 most variable genes, `scale_features = FALSE` and a perplexity of 3. The Laplacian Eigenmaps embedding was found with `embeddr`, again using the top 195 most variable genes, a euclidean distance metric and 30 nearest neighbours.

The GPLVM was fitted on the PCA embedding (as in original publication) using `pseudogp`. The MCMC chain was initialised from the first component of the PCA of the representation and smoothing hyperparameters of $\gamma_\alpha = 8$, $\gamma_\beta = 2$.

1.4 Differential expression analysis

Two types of differential expression analysis are considered - smoothing spline regression with Tobit censored values and the switch-like sigmoidal differential expression presented here.

For standard differential expression smoothing-spline regression models were fit using B-spline basis functions of degree 3 (cubic) and 3 degrees of freedom using the `splines` package and Tobit censoring using the `AER` package, with a lower threshold of 0.1 for all 3 datasets and hypothesis testing performed identically to [?].

1.4.1 Switch-like differential expression

Non-zero-inflated model We propose a differential gene expression model of the form

$$y_{ij}(t_j) = \mathcal{N}(\mu_i(t_j),\ \sigma_i^2) \tag{1}$$

where y_{ij} is the $\log_2$ gene expression of gene i in cell j at pseudotime t_j, and

$$\mu_i(t_j) = \begin{cases} \mu_i^{(0)}, & \text{if gene } i \text{ not differentially expressed} \\ \frac{2\mu_i^{(0)}}{1+\exp\left(-k_i(t_j - t_i^{(0)})\right)}, & \text{if gene } i \text{ differentially expressed} \end{cases} \tag{2}$$

The case of a gene not being differentially expressed is a nested model of the differential expression case found by setting $k = 0$. Consequently we can use a likelihood ratio test with no differential expression as the null hypothesis and differential expression as the alternative and twice the difference in their log-likelihoods will form a χ^2 test statistic with 2 degrees of freedom.

Zero-inflated model Single-cell gene expression data is known to exhibit dropout, where lowly expressed genes register as a 0 count due to reverse-transcription failure. We base the zero-inflated component of the model largely on that described in [?], so our model becomes

$$\begin{aligned} x_{ij} &\sim \mathcal{N}(\mu_i(t_j), \sigma_i) \\ h_{ij}|x_{ij} &\sim \text{Bernoulli}(\exp(-\lambda x_{ij}^2)) \\ y_{ij} &= \begin{cases} x_{ij}, & \text{if } h_{ij} = 0 \\ 0, & \text{if } h_{ij} = 1 \end{cases} \end{aligned} \tag{3}$$

The non-zero-inflated model was fitted using maximum likelihood while the zero-inflated model using the EM algorithm.

Inference In both cases the derivatives have no analytical solution so the MLEs were found numerically using L-BFGS-B optimisation. Inference in the zero-inflated case was performed using expectation-maximisation (EM), largely based on [**?**] with a couple of differences, such as the E-step for x_{ij} being given by $x_{ij}^{t+1} = \frac{\mu_{ij}}{1+2\sigma_{ij}^2\lambda}$. There are also a couple of numerical issues that can cause singularities in the derivatives. One term in the derivative of the log-likelihood includes the term $\ln(1 - \exp(-\lambda x^2))$ which for very small λx^2 evaluates to $-\infty$. Here we use the series expansion which approximates $\ln(1 - \exp(-\lambda x^2)) \approx \ln(\lambda x^2)$. Similarly there is a term involving $\frac{x^2}{\exp(\lambda x^2)-1}$ for which we use the approximation $\frac{x^2}{\exp(\lambda x^2)-1} \approx \frac{1}{\lambda} - \frac{x^2}{2}$. We initialise the parameters as follows: $\mu_i^{(0)} = \frac{1}{n_{genes}} \sum_j y_{ij}$, $t_i^0 = \text{median}(t_j)$, $\sigma_i^2 = \text{Var}_i(y_{ij})$. k_i is initialised by regressing the vector y_{ij} (for a given i) off t_j and using the slope as the initial estimate, ensuring the sign of k will be correct and the optimisation won't get stuck in a local minimum. In the zero-inflated models, λ is initialised to 1.
