# Supplementary figures and images for: Order Under Uncertainty: Robust Differential Expression Analysis Using Probabilistic Models for Pseudotime Inference
Source: PLoS Comput Biol. 2016 Nov 21;12(11):e1005212. doi: 10.1371/journal.pcbi.1005212 (PMC5117567; doi:10.1371/journal.pcbi.1005212)

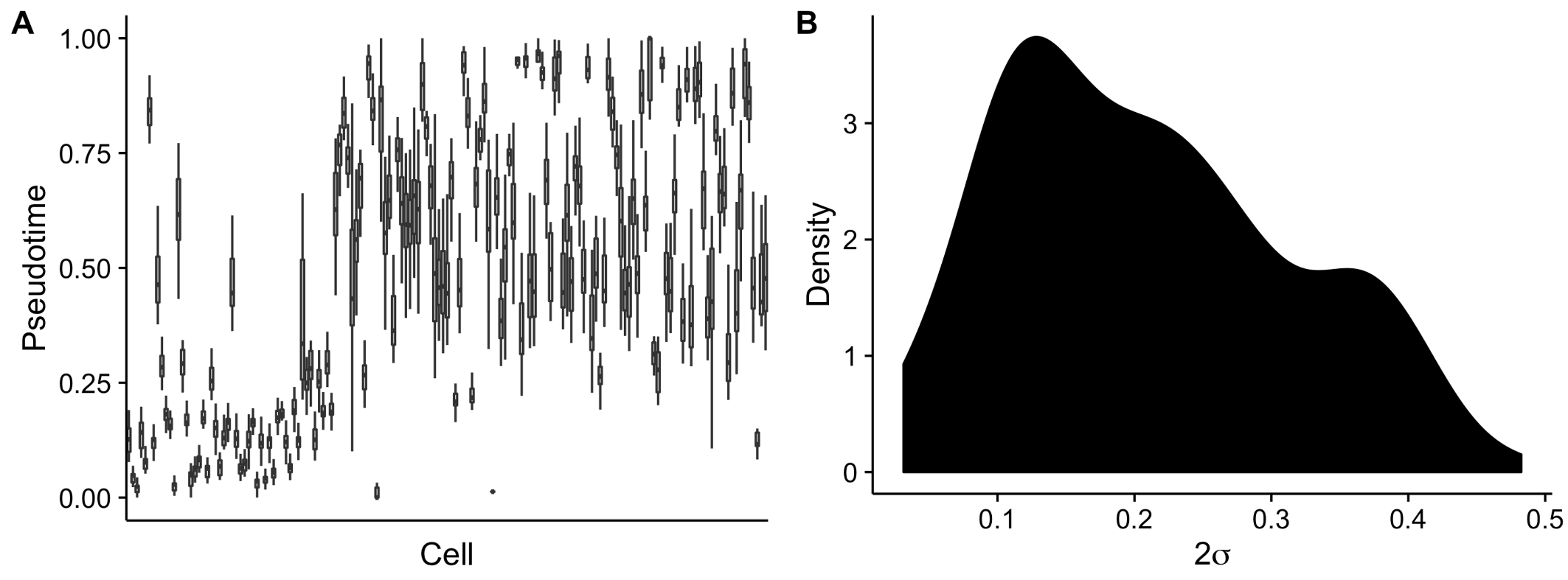

Supplement: S1 Fig — 80% of cells were subsampled from the Laplacian Eigenmaps representation 30 times and the pseudotime refitted using Monocle’s MST approach. A Boxplots of resamples across all cells. Upper and lower whiskers extend to the highest and lowest values within 1.5 times the interquartile range. B The 2σ interval was then computed for the pseudotime of each cell, which varies from as low as close to 0 up to almost half the pseudotime interval (0.5). (TIF) [file pcbi.1005212.s002.tif]

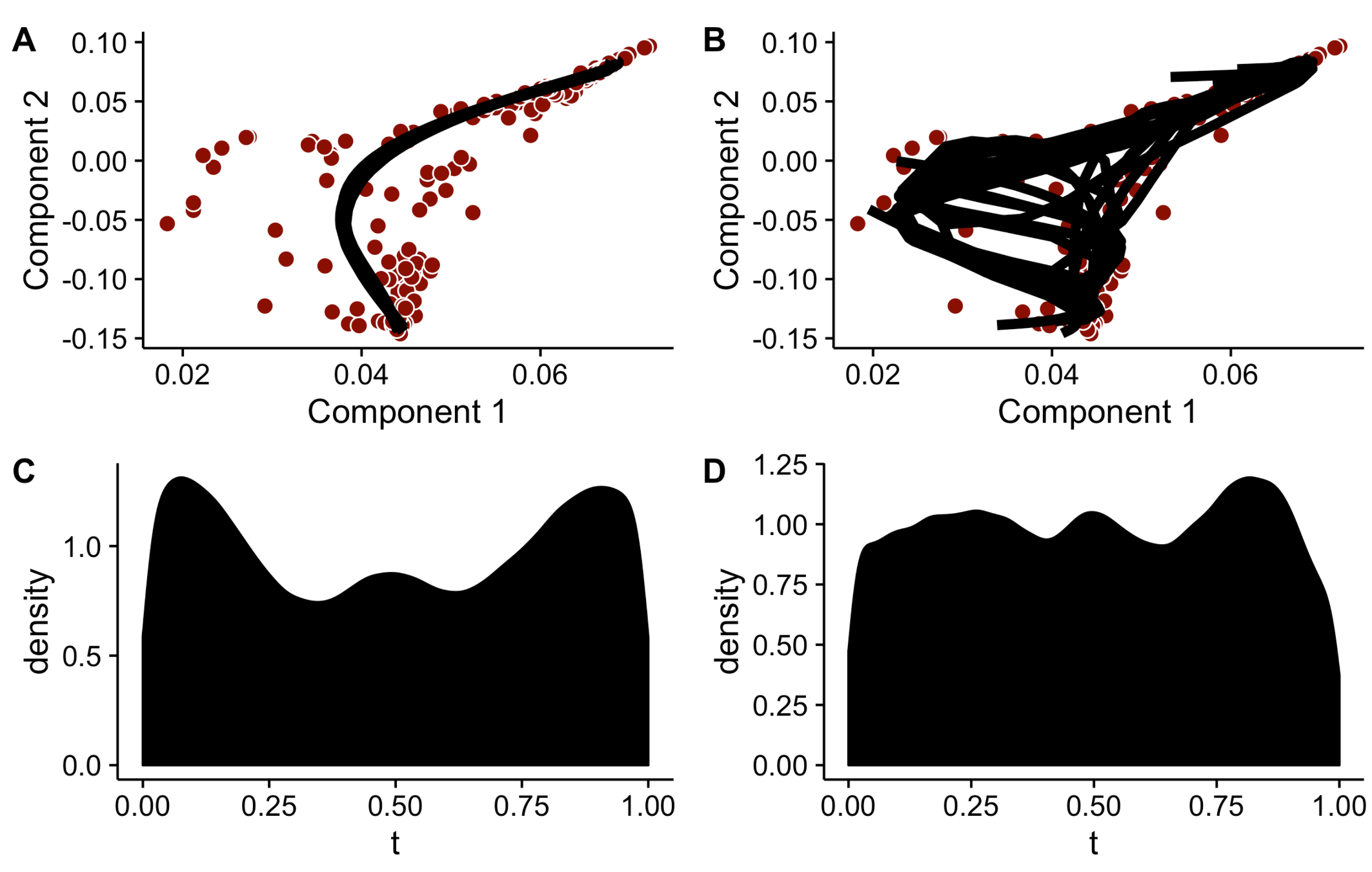

Supplement: S2 Fig — GPLVM trajectories were fit on the Trapnell dataset using either a high level of shrinkage in A & C (γα = 35, γβ = 5) or using a low level of shrinkage in B & D (γα = 3, γβ = 1) for 10 separate MCMC chains. It can be seen that the trajectories consistently fit using high levels of shrinkage, implying this is required to have a well-defined posterior as opposed to a ‘lumpy’ posterior with many local maxima using low levels of shrinkage. C & D show the posterior densities for a randomly chosen cell (number 100) using the two different shrinkage regimes as defined above. (TIF) [file pcbi.1005212.s003.tif]

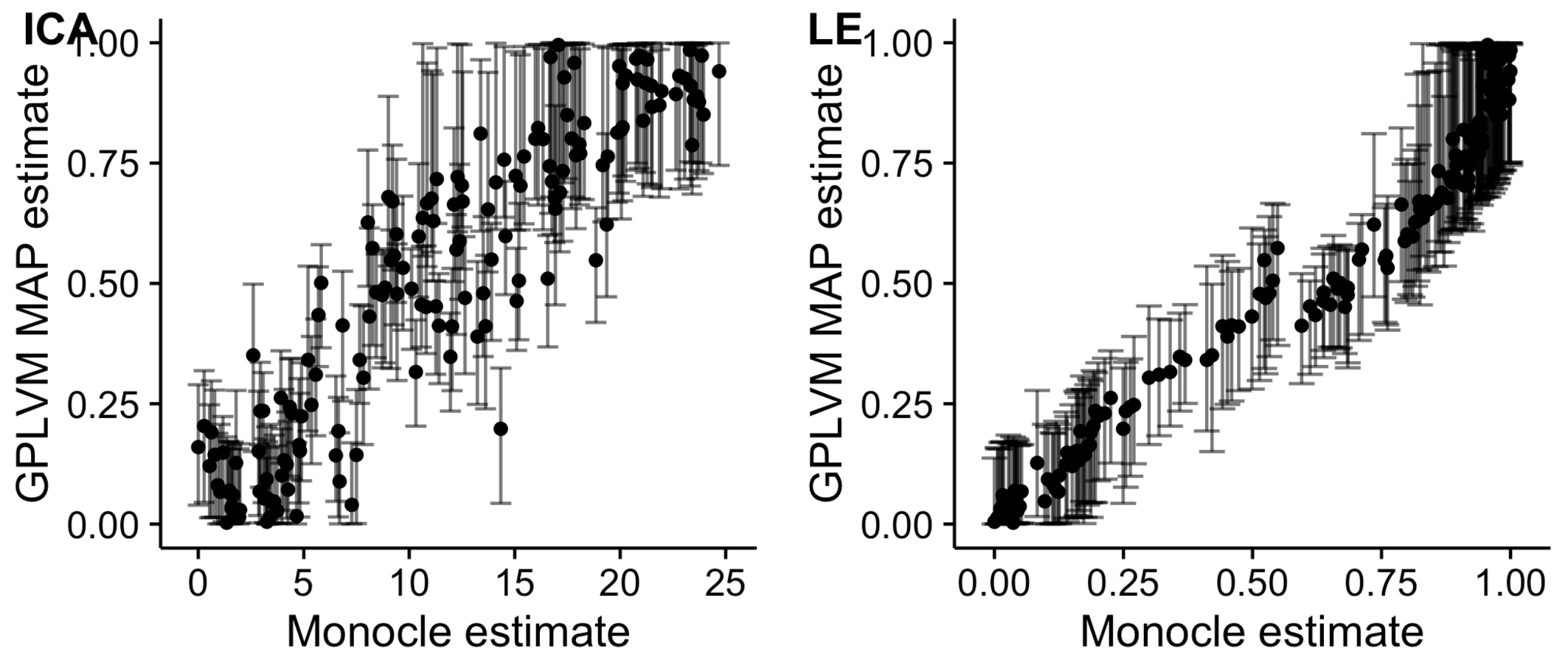

Supplement: S3 Fig — Left: Monocle ordering found using ICA on 500 most variable genes. Right: Monocle orderings found using a Laplacian Eigenmaps embedding as described above. Error bars show the 95% HPD credible interval. (TIF) [file pcbi.1005212.s004.tif]
