# Supplementary material for: Correction: Order Under Uncertainty: Robust Differential Expression Analysis Using Probabilistic Models for Pseudotime Inference
Source: PLoS Comput Biol. 2017 Apr 11;13(4):e1005477. doi: 10.1371/journal.pcbi.1005477 (PMC5388462; doi:10.1371/journal.pcbi.1005477)
Supplement: S1 File — (PDF) [file pcbi.1005477.s001.pdf]

PLOS COMPUTATIONAL BIOLOGY

RESEARCH ARTICLE

Order Under Uncertainty: Robust Differential Expression Analysis Using Probabilistic Models for Pseudotime Inference

Kieran R. Campbell[1,2], **Christopher Yau**[2,3]*

1 Department of Physiology, Anatomy and Genetics, University of Oxford, Oxford, United Kingdom, **2** Wellcome Trust Centre for Human Genetics, University of Oxford, Oxford, United Kingdom, **3** Department of Statistics, University of Oxford, Oxford, United Kingdom

* cyau@well.ox.ac.uk

CrossMark
click for updates

OPEN ACCESS

Citation: Campbell KR, Yau C (2016) Order Under Uncertainty: Robust Differential Expression Analysis Using Probabilistic Models for Pseudotime Inference. PLoS Comput Biol 12(11): e1005212. doi:10.1371/journal.pcbi.1005212

Editor: Magnus Rattray, University of Manchester, UNITED KINGDOM

Received: April 21, 2016

Accepted: October 13, 2016

Published: November 21, 2016

Copyright: © 2016 Campbell, Yau. This is an open access article distributed under the terms of the Creative Commons Attribution License, which permits unrestricted use, distribution, and reproduction in any medium, provided the original author and source are credited.

Data Availability Statement: Our software is available as an R package available at Github: http://www.github.com/kieranrcampbell/pseudogp.

Funding: KRC is supported by a UK Medical Research Council funded doctoral studentship. CY is supported by a UK Medical Research Council New Investigator Research Grant (Ref. No. MR/L001411/1), the Wellcome Trust Core Award Grant Number 090532/Z/09/Z, the John Fell Oxford University Press (OUP) Research Fund and the Li Ka Shing Foundation via a Oxford-Stanford Big Data in Human Health Seed Grant. The funders had no role in study design, data collection and

Abstract

Single cell gene expression profiling can be used to quantify transcriptional dynamics in temporal processes, such as cell differentiation, using computational methods to label each cell with a 'pseudotime' where true time series experimentation is too difficult to perform. However, owing to the high variability in gene expression between individual cells, there is an inherent uncertainty in the precise temporal ordering of the cells. Pre-existing methods for pseudotime estimation have predominantly given point estimates precluding a rigorous analysis of the implications of uncertainty. We use probabilistic modelling techniques to quantify pseudotime uncertainty and propagate this into downstream differential expression analysis. We demonstrate that reliance on a point estimate of pseudotime can lead to inflated false discovery rates and that probabilistic approaches provide greater robustness and measures of the temporal resolution that can be obtained from pseudotime inference.

Author Summary

Understanding the "cellular programming" that controls fundamental, dynamic biological processes is important for determining normal cellular function and potential perturbations that might give rise to physiological disorders. Ideally, investigations would employ time series experiments to periodically measure the properties of each cell. This would allow us to understand the sequence of gene (in)activations that constitute the program being followed. In practice, such experiments can be difficult to perform as cellular activity may be asynchronous with each cell occupying a different phase of the process of interested. Furthermore, the unbiased measurement of all transcripts or proteins requires the cells to be captured and lysed precluding the continued monitoring of that cell. In the absence of the ability to conduct true time series experiments, pseudotime algorithms exploit the asynchronous cellular nature of these systems to mathematically assign a "pseudotime" to each cell based on its molecular profile allowing the cells to be aligned and the sequence of gene activation events retrospectively inferred. Existing approaches

analysis, decision to publish, or preparation of the manuscript.

Competing Interests: The authors have declared that no competing interests exist.

predominantly use deterministic methods that ignore the statistical uncertainties associated with the problem. This paper demonstrates that this statistical uncertainty limits the temporal resolution that can be extracted from static snapshots of cell expression profiles and can also detrimentally affect downstream analysis.

Introduction

The emergence of high-throughput single cell genomics as a tool for the precision study of biological systems [1–4] has given rise to a variety of novel computational and statistical modelling challenges [5, 6]. One particular area of interest has been the study of transcriptional dynamics in temporal processes, such as cell differentiation or proliferation [7, 8], in order to understand the coordinated changes in transcription programming that underlie these processes. In the study of such systems, practical experimental designs that can allow the collection of real time series data maybe difficult or impossible to achieve. Instead, investigators have adopted computational methods to identify temporal signatures and trends from unordered genomic profiles of single cells, a process known as *pseudotemporal ordering* [9–19]. Computational approaches for this problem were first tackled in the context of gene expression microarray analysis of bulk cell populations [20–22] but the recent availability of single cell technology overcomes the limitations of measuring population averaged signals in bulk analyses.

Pseudotemporal ordering of whole transcriptome profiles of single cells with unsupervised computational methods has an advantage over flow cytometry-based assays in that it does not rely on *a priori* knowledge of marker genes. The principle underlying these methods is that each single cell RNA sequencing experiment constitutes a time series in which each cell represents a distinct time point along a continuum representing the underlying degree of temporal progress (Fig 1A). During the single cell capture process, the *true* temporal label that identifies the stage of the cell is lost (Fig 1B) and these parameters become latent, unobserved quantities that must be statistically inferred from the collection of single cell expression profiles (Fig 1C). Importantly, absolute physical time information will in general be irretrievably lost and it is only possible to assign a "pseudotime" for each cell that provides a relative quantitative measure of progression. Consequently, whilst the correspondence between physical and pseudotime ordering maybe conserved, the pseudotimes themselves are not necessarily calibrated to actual physical times. Pseudotime estimation can potentially be used to recapitulate temporal resolution in an experiment that does not explicitly capture labelled time series data. The pseudotimes could then be used to identify genes that are differentially expressed across pseudotime (Fig 1D) providing insight into the evolution of transcription programming.

Practically, current methods for pseudotime inference proceed via a multi-step process which we describe throughout using gene expression data as the focus of our discussion. First, gene selection and dimensionality reduction techniques are applied to compress the information held in the high-dimensional gene expression profiles to a small number of dimensions (typically two or three for simplicity of visualisation). The identification of an appropriate dimensionality reduction technique is a *subjective* choice and a number of methods have been adopted such as Principal and Independent Components Analysis (P/ICA) and highly non-linear techniques such as diffusion maps [17, 23] or stochastic neighbourhood embedding (SNE) [24–26]. This choice is guided by whether the dimensionality reduction procedure is able to identify a suitable low-dimensional embedding of the data that contains a relatively smooth trajectory that might plausibly correspond to the temporal process under investigation.

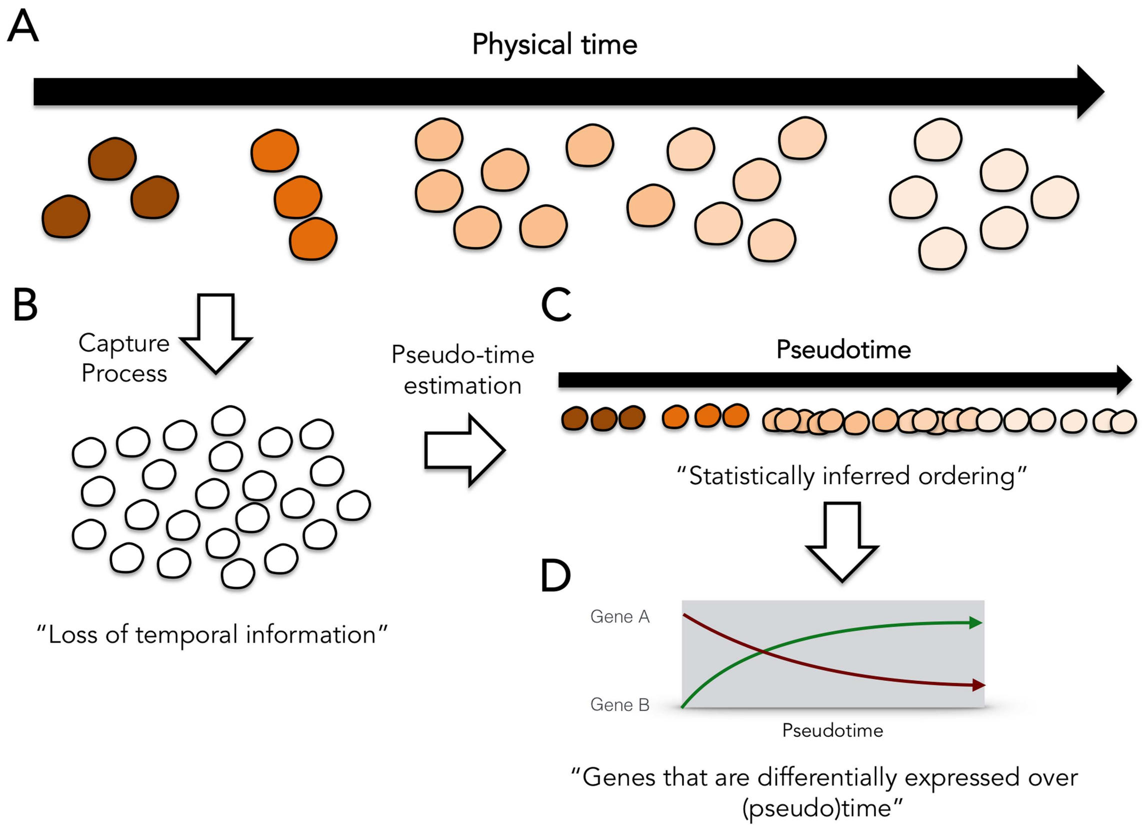

Fig 1. The single cell pseudotime estimation problem. (A) Single cells at different stages of a temporal process. (B) The temporal labelling information is lost during single cell capture. (C) Statistical pseudotime estimation algorithms attempt to reconstruct the relative temporal ordering of the cells but cannot fully reproduce physical time. (D) The pseudotime estimates can be used to identify genes that are differentially expressed over (pseudo)time.

doi:10.1371/journal.pcbi.1005212.g001

Next, the pseudotime trajectory of the cells in this low-dimensional embedding is characterised. In Monocle [12] this is achieved by the construction of a minimum spanning tree (MST) joining all cells. The diameter of the MST provides the main trajectory along which pseudotime is measured. Related graph-based techniques (Wanderlust) have also been used to characterise temporal processes from single cell mass cytometry data [10]. In SCUBA [11] the trajectory itself is directly modelled using principal curves [27] and pseudotime is assigned to each cell by projecting its location in the low-dimensional embedding on to the principal curve. The estimated pseudotimes can then be used to order the cells and to assess differential expression of genes across pseudotime. Note that in the diffusion pseudotime framework [17], all the diffusion components are used in the random-walk pseudotime model and there is no strict dimensionality reduction step. However, the derivation of the diffusion maps does lead to the compression of information into the first few diffusion components which is what enables successful visualisation [23].

A limitation of these approaches is that they provide only a single *point estimate* of pseudotimes concealing the full impact of variability and technical noise. As a consequence, the statistical uncertainty in the pseudotimes is not propagated to downstream analyses precluding a thorough treatment of stability. To date, the impact of this pseudotime uncertainty has not been explored and its implications are unknown as the methods applied typically do not possess a probabilistic interpretation. However, we can examine the stability of the pseudotime

estimates by taking multiple random subsets of a dataset and re-estimating the pseudotimes for each subset. For example, we have found that the pseudotime assigned to the same cell can vary considerably across random subsets in Monocle (details given in S1 Text and S1 Fig).

In order to address pseudotime uncertainty in a formal and coherent framework, probabilistic approaches using Gaussian Process Latent Variable Models (GPLVM) have been used recently as non-parametric models of pseudotime trajectories [14, 28, 29]. These provide an explicit model of pseudotimes as latent embedded one-dimensional variables. These models can be fitted within a Bayesian statistical framework using priors on the pseudotimes [14], deterministic optimisation methods for approximate inference [29] or Markov Chain Monte Carlo (MCMC) simulations allowing full posterior uncertainty in the pseudotimes to be determined [28]. In this article we adopt this framework based to assess the impact of pseudotime uncertainty on downstream differential analyses. We will show that pseudotime uncertainty can be non-negligible and when propagated to downstream analysis may considerably inflate false discovery rates. We demonstrate that there exists a limit to the degree of recoverable temporal resolution, due to intrinsic variability in the data, with which we can make statements such as "this cell precedes another". Finally, we propose a simple means of accounting for the different possible choices of reduced dimension data embeddings. We demonstrate that, given sensible choices of low-dimensional representations, these can be combined to produce more robust pseudotime estimates. Overall, we outline a modelling and analytical strategy to produce more stable pseudotime based differential expression analysis.

Methods

Statistical model for probabilistic pseudotime

The hierarchical model specification for the Gaussian Process Latent Variable model is described as follows:

$$\begin{aligned}
\gamma &\sim \text{Gamma}(\gamma_\alpha, \gamma_\beta), \\
\lambda_j &\sim \text{Exp}(\gamma),\ j = 1, \ldots, P, \\
\sigma_j^2 &\sim \text{InvGamma}(\alpha, \beta),\ j = 1, \ldots, P, \\
t_i &\sim \text{TruncNormal}_{[0,1)}(\mu_t, \sigma_t^2),\ i = 1, \ldots, N, \\
\boldsymbol{\Sigma} &= \text{diag}(\sigma_1^2, \ldots, \sigma_P^2) \\
K^{(j)}(t, t') &= \exp\left(-\lambda_j (t - t')^2\right),\ j = 1, \ldots, P, \\
\mu_j &\sim \text{GP}(0, K^{(j)}),\ j = 1, \ldots, P, \\
\mathbf{x}_i &\sim \text{MultiNorm}(\boldsymbol{\mu}(t_i), \boldsymbol{\Sigma}),\ i = 1, \ldots, N.
\end{aligned} \tag{1}$$

where $\mathbf{x}_i$ is the P-dimensional input of cell i (of N) found by performing dimensionality reduction on the entire gene set (for our experiments $P = 2$ following previous studies). The observed data is distributed according to a multivariate normal distribution with mean function $\boldsymbol{\mu}$ and a diagonal noise covariance matrix Σ. The prior over the mean function $\boldsymbol{\mu}$ in each dimension is given by a Gaussian Process with zero mean and covariance function K given by a standard double exponential kernel. The latent pseudotimes $t_1, \ldots, t_N$ are drawn from a truncated Normal distribution on the range [0, 1). Under this model $|\boldsymbol{\lambda}|$ can be thought of as the arc-length of the pseudotime trajectories, so applying larger levels of shrinkage to it will result in smoother trajectories passing through the point space. This shrinkage is ultimately controlled by the gamma hyperprior on γ, whose mean and variance are given by $\frac{\gamma_\alpha}{\gamma_\beta}$ and $\frac{\gamma_\alpha}{\gamma_\beta^2}$

respectively. Therefore, adjusting these parameters allows curves to match prior smoothness expectations provided by plotting marker genes.

The hyperparameters γ_α, γ_β, α, β, μ_t and σ_t^2 are fixed and values for specific experiments for given in S1 Text. Inference was performed using the Stan probabilistic programming language [30] and our implementation is available as an `R` package at http://www.github.com/kieranrcampbell/pseudogp.

Sigmoidal model for switch-like gene (in)activation behaviours across pseudotime

We detail the mathematical specification of the sigmoidal switch model below. Let y_{ij} denote the $\log_2$ gene expression of gene i in cell j at pseudotime t_j then

$$y_{ij}(t_j) \sim \text{Norm}(\mu_i(t_j),\ \sigma_i^2) \tag{2}$$

where

$$\mu_i(t_j) = \begin{cases} \mu_i^{(0)}, & \text{if gene } i \text{ not differentially expressed,} \\ \dfrac{2\mu_i^{(0)}}{1+\exp\left(-k_i(t_j - t_i^{(0)})\right)}, & \text{if gene } i \text{ differentially expressed.} \end{cases} \tag{3}$$

Under this model the parameter k_i can be thought of as an activation 'strength' relating to how quickly a gene switches on or off, while $t_i^{(0)}$ relates to the pseudotime at which the gene switches on or off.

The case of a gene not being differentially expressed is a nested model of the differential expression case found by setting $k = 0$. Consequently we can use a likelihood ratio test with no differential expression as the null hypothesis and differential expression as the alternative and twice the difference in their log-likelihoods will form a χ^2 test statistic with 2 degrees of freedom. The maximum likelihood estimates of the parameters under the differential expression model have no analytical solution so L-BFGS-B optimisation was used (implemented in the `R` package `switchde`, http://github.com/kieranrcampbell/switchde). The dropout model from ZIFA [31] can be incorporated into the model and details are given in S1 Text.

Results

Probabilistic pseudotime inference using Gaussian Process Latent Variable Models

We first provide a brief overview of the Gaussian Process Latent Variable Model [32]. The GPLVM uses a Gaussian Process to define a stochastic mapping between a low-dimensional latent space to a (typically) higher dimensional observation space. A Gaussian Process is characterised by a mean function describing the expected mapping between the latent and observation spaces and a covariance function that describes the covariance between the mapping function evaluated at any two arbitrary latent positions. The covariance function therefore acts to control the second-order statistics of the Gaussian Process and suitable choices can be designed to encourage properties such as smoothness, periodicity or other second-order features.

For this application, the latent space is one-dimensional, describing pseudotime progression whilst the observations are the reduced dimensionality representations of the single cell expression data. We will use Bayesian inference to characterise the joint posterior distribution $p(\mathbf{t}|\mathbf{X})$ of the pseudotimes $\mathbf{t} = \{t_1, \ldots, t_n\}$ given the expression data $\mathbf{X} = \{\mathrm{x}_1, \ldots, \mathrm{x}_n\}$ for n single

cells. As the integrals involved are mathematically intractable, we will use Markov Chain Monte Carlo simulations to obtain a numerical approximation to the posterior by drawing samples from the posterior distribution. Each sample corresponds to one possible trajectory *and* ordering for the cells with the set of samples providing an approximate distribution of pseudotimes. The pseudotime values are between measured 0 and 1 where a value of 0 corresponds to one end state of the temporal process and a value 1 to the other. In this work we focus only on non-bifurcating processes. Fig 2 gives a diagrammatic representation of our proposed workflow and a more detailed model descriptions is given in Methods.

Sources of uncertainty in pseudotime inference

We applied our probabilistic pseudotime inference to three published single-cell RNA-seq datasets of differentiating cells: myoblasts in Trapnell et al. (2014) [12], hippocampal quiescent neural stem cells in Shin et al. (2015) [16] and sensory epithelia from the inner ear in Burns et al. (2015) [33]. For the Trapnell and Shin datasets we used Laplacian Eigenmaps [34] for dimensionality reduction prior to pseudotime inference, while for the Burns dataset we used the PCA representation of the cells from the original publication (for a detailed description of our analysis, see S1 Text). These particular choices of reduced dimensionality representations gave visually plausible trajectory paths in two dimensions.

An implicit assumption in pseudotime estimation is that proximity in pseudotime should reflect proximity in the observation or data space. That is, two cells with similar pseudotime assignments should have similar gene expression profiles but, in practice, cell-to-cell variability and technical noise means that the location of the cells in the observation space will be variable even if they truly do have the same pseudotime. We plotted posterior mean pseudotime trajectories for the three datasets learned using the GPLVM in Fig 3A–3C and the posterior predictive data distribution $p(\mathbf{X}^*|\mathbf{X})$. The posterior predictive data distribution gives an indication of where *future* data points might occur given the existing data. Notice that for all three data sets, this distribution can be quite diffuse. This is due to a combination of actual cell-to-cell expression variability, manifesting as a spread of data points around the mean trajectory, but also model misspecification (the difference between what our "assumed" model and the "true" but unknown data generating mechanism).

It is interesting to discuss the latter point as it is an issue that is often not adequately addressed or fully acknowledged in the literature. The GPLVM applied assumes a homoscedastic noise distribution which is uniform along the pseudotime trajectory. However, it is clear

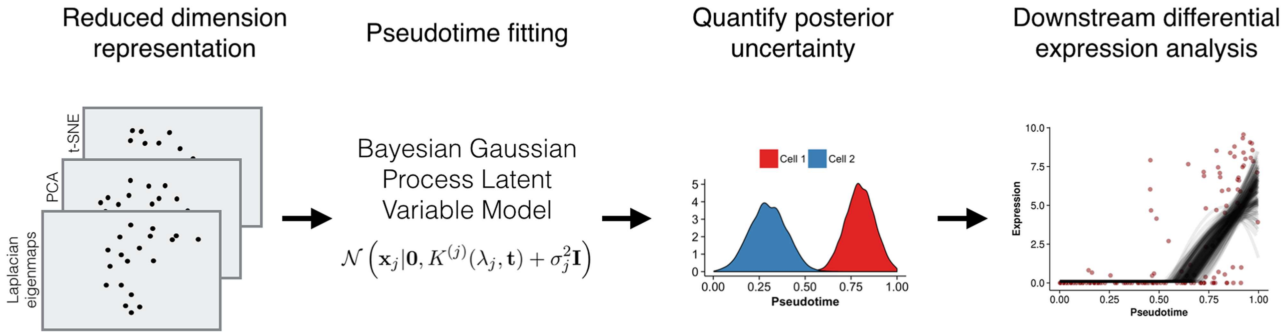

Fig 2. Workflow for fitting Bayesian Gaussian Process Latent Variable Model pseudotime models. Reduced-dimension representations of the gene expression data (from Laplacian eigenmaps, PCA or t-SNE) are created. The pseudotime can be fitted using one or more low dimensional representations of the data. Posterior samples of pseudotimes are drawn from a Bayesian GPLVM and these are used to obtain alternative pseudotime estimates. Downstream differential analyses can be performed on the posterior samples to characterise the robustness with respect to variation in pseudotime estimates.

doi:10.1371/journal.pcbi.1005212.g002

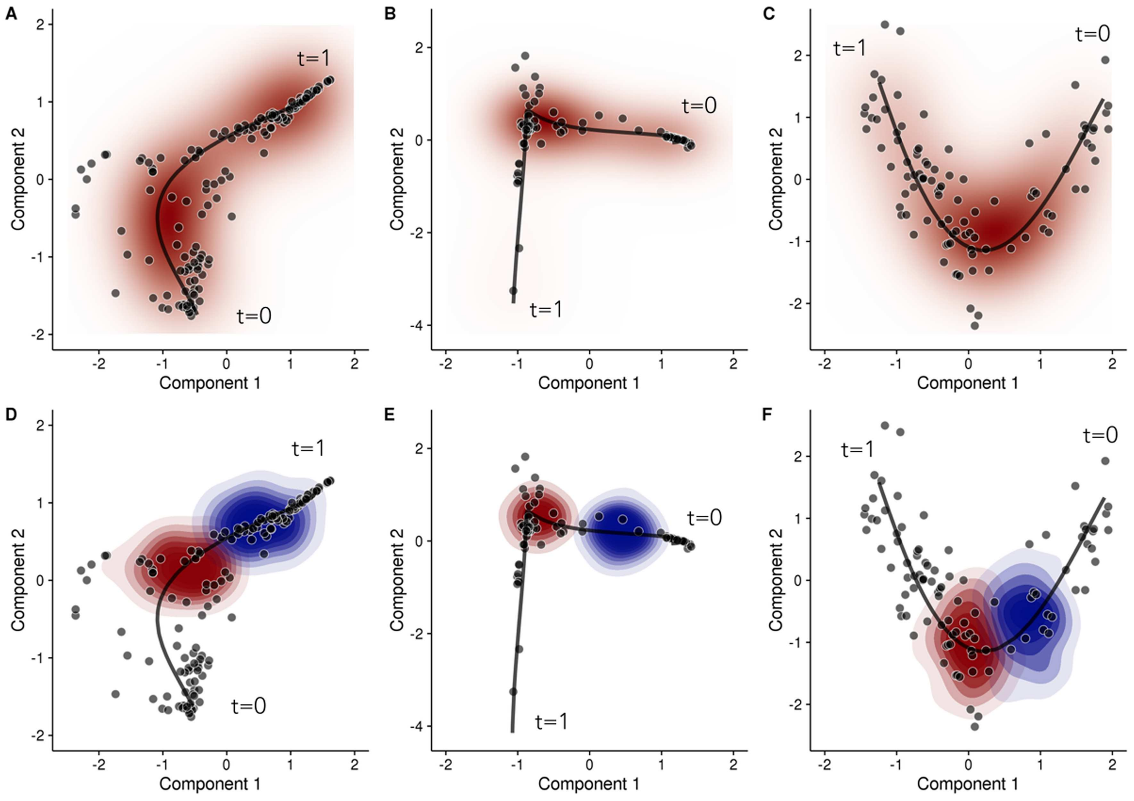

Fig 3. Posterior pseudotime trajectories for three single-cell RNA-seq datasets. Posterior pseudotime trajectories shown in a two-dimensional reduced representation space for (left) a Laplacian eigenmaps representation of Trapnell et al. (2014) [12], (centre) Laplacian eigenmaps representation of Burns et al. (2015) [33] and (right) PCA representation of Shin et al. (2015) [16]. Each point represents a cell and the black line represents the mean pseudotime trajectory. Plots **(A-C)** shows the overall posterior predictive data density (red) whilst **(D-F)** shows the conditional posterior predictive data density for $t = 0.5$ (red) and $t = 0.7$ (blue).

doi:10.1371/journal.pcbi.1005212.g003

that the variability of the data points can change along the trajectory and a heteroscedastic (non-uniform) noise model may be more appropriate in certain scenarios. Unfortunately, whilst models of heteroscedastic noise processes can be applied [35], these typically severely complicate the statistical inference and require a model of how the variability changes over pseudotime which is likely to be unknown. The important point here is that the posterior probability calculations are always calibrated with respect to a given model. The better the model represents the true data generating mechanism, the better calibrated the probabilities. Model misspecification can also contribute to posterior uncertainty in inferred parameters.

Returning to the intrinsic cell-to-cell variability, we next considered the conditional posterior predictive data distributions $p(\mathbf{X}^*|t^*,\mathbf{X})$ which are shown in Fig 3D–3F. These distributions show the possible distribution of future data points given the existing data *and* a theoretical pseudotime t^* and, in this example, we condition on pseudotimes $t^* = 0.5$ and $t^* = 0.7$. Although the two pseudotimes differ by a magnitude of 0.2, the conditional predictive distributions are very close or overlapping. This means that cells with pseudotimes of 0.5 or 0.7 could have given rise to data point occupying these overlapping regions. This variability is what ultimately limits the temporal resolution that can be obtained.

It is important to note that the posterior mean trajectories correspond to certain *a priori* or subjective smoothness assumptions (specified as hyperparameters in the model specification) which dedicate the curvature properties of the trajectory. Fig 4 shows three alternative posterior

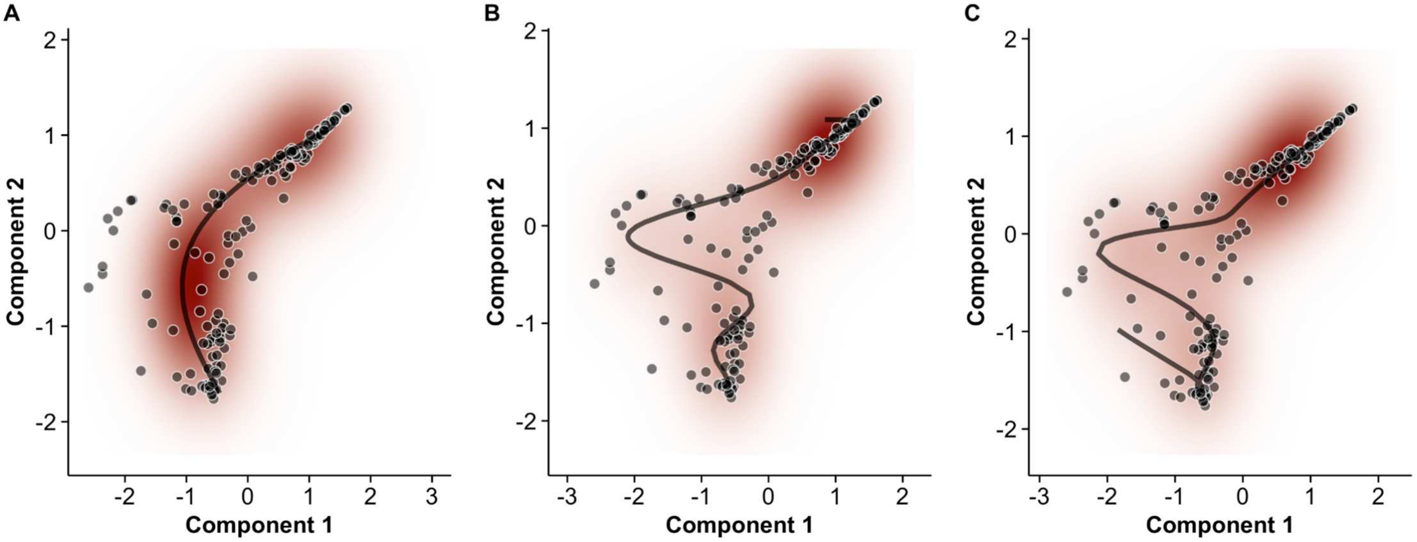

Fig 4. Effect of prior expectations on pseudotime trajectories. The prior probability distribution (defined in terms of hyperparameters (γ_α, γ_β) in our model) on the expected smoothness of pseudotime trajectories can fundamentally change the inferred progression path. Examples shown using the data of Trapnell et al. (2014) [12]. Red—shows the density of the posterior predictive data distribution. Black—shows the mean pseudotime trajectory. Shrinkage hyperparameters (γ_α, γ_β) of (30, 5), (5, 1) and (3, 1) were used for **A**, **B** and **C** respectively.

doi:10.1371/journal.pcbi.1005212.g004

mean pseudotime trajectories for the Trapnell data based on different hyperparameters settings for the GPLVM. In a truly unsupervised scenario all three paths could be plausible as we would have little information to inform us about the true shape of the trajectory. This would become an additional source of uncertainty in the pseudotime estimates. However, we favoured hyper-parameter settings that gave rise to well-defined (unimodal) posterior distributions that resulted in multiple independent Markov Chain Monte Carlo runs converging to the same mean trajectory rather than settings that give rise to a "lumpy" posterior distribution with many local modes corresponding to different interpretations of the data (see S2 Fig). Later on, when we consider inference using multiple representations, the ability to specify a wider choice of trajectories is useful as we will demonstrate how the correspondence between pseudotime trajectories in different reduced dimension representations is not always obvious from a visual analysis.

We next examined the posterior distributions in pseudotime assignment for four cells from the Trapnell dataset in Fig 5A. Uncertainty in the estimate of pseudotime is assessed using the highest probability density (HPD) credible interval (CI), the Bayesian equivalent of the confidence interval. The 95% pseudotime CI typically covers around one quarter of the trajectory, suggesting that pseudotemporal orderings of single-cells can potentially only resolve a cell's place within a trajectory to a coarse estimate (e.g. 'beginning', 'middle' or 'end') and do not necessarily dramatically increase the temporal resolution of the data. One immediate consequence of this is that it is unlikely that we can make definite statements such as whether one cell comes exactly before or after another. This is illustrated in Fig 5B–5D which displays the estimated pseudotime uncertainty for all three datasets. In all the datasets, the general progression is apparent, but the precise ordering of the cells has a non-trivial degree of ambiguity.

Failure to account for pseudotime uncertainty leads to increased false discovery rates

The previous section addressed the sources of statistical uncertainty in the pseudotimes. We next explored the impact of pseudotime uncertainty on downstream analysis. Specifically, we

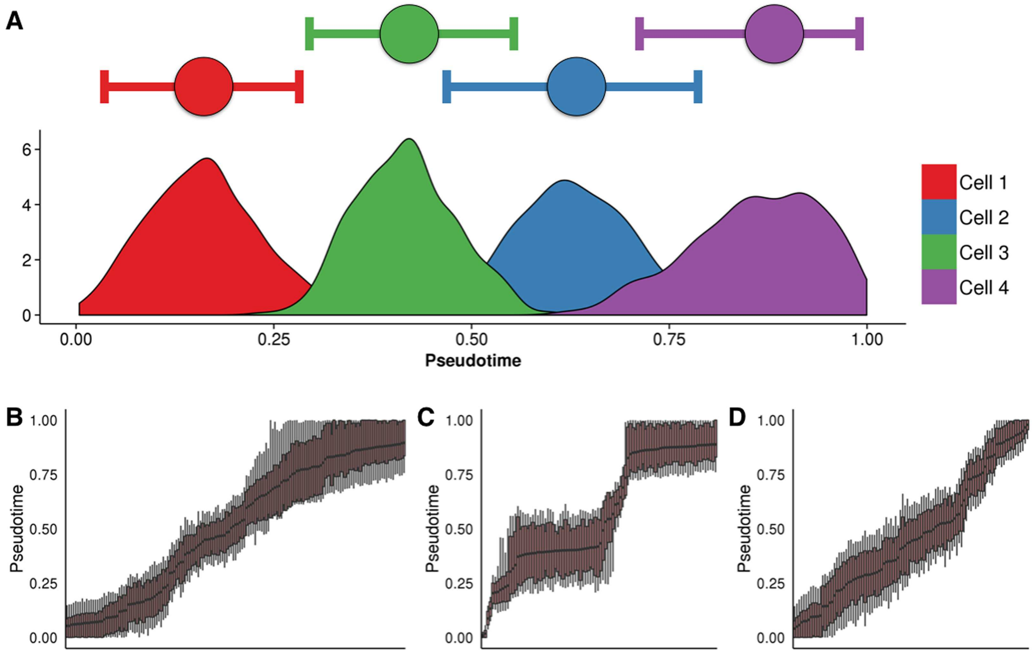

Fig 5. Posterior uncertainty in pseudotime trajectories. (A) Posterior uncertainty in pseudotimes for four randomly selected cells from the Trapnell et al. (2014) dataset. Horizontal bars represent the 95% highest probability density (HPD) credible interval (CI), which typically covers around a quarter of the pseudotime trajectory. **(B-D)** Boxplots showing the posterior uncertainty for each cell from the Trapnell et al. (2014) datasets. The edges of the boxes and tails correspond to the 75% and 95% HPD-CIs respectively.

doi:10.1371/journal.pcbi.1005212.g005

focused on the identification of genes that are differentially expressed across pseudotime. Typically, these analyses involve regression models that assume the input variables (the pseudotimes) are both fixed and certain but, with our probabilistic model, we can use the posterior samples from our Bayesian model to refit the regression model to each pseudotime estimate. In doing so we can examine which genes are called as significant in each of the posterior samples and assess the stability of the differential expression analysis to pseudotime uncertainty by recording how frequently genes are designated as significant across the posterior samples. This allowed us to re-estimate the false discovery rate (FDR) fully accounting for the variability in pseudotime. As there are a multitude of sources of uncertainty on top of this (such as biological and technical variability) this allows us to put a lower bound on the FDR of such analyses in general.

Precisely, we fitted the tobit regression model from [12] for each gene for each sample from the posterior pseudotime distribution, giving us a per-gene set of false-discovery-rate-corrected *Q*-values. We then compared the proportion of times a gene is called as differentially expressed (5% FDR) across all pseudotime samples to the *Q*-value using a point pseudotime estimate based on the maximum *a posteriori* (or MAP) estimate. We reasoned that if a gene is truly differentially expressed then such expression will be robust to the underlying uncertainty in the ordering. Note for comparison, our MAP estimates with the GPLVM correlate strongly with Monocle derived pseudotime point estimates (see S3 Fig).

Fig 6(A) and 6(B) shows two analyses for two illustrative genes (ITGAE and ID2) in the Trapnell data set. Using the MAP pseudotime estimates, differential expression analysis of ITGAE over pseudotime attained a *q*-value of 0.02. However, the gene was only called significant in only 9% of posterior pseudotime samples with a median *q*-value of 0.32. In contrast,

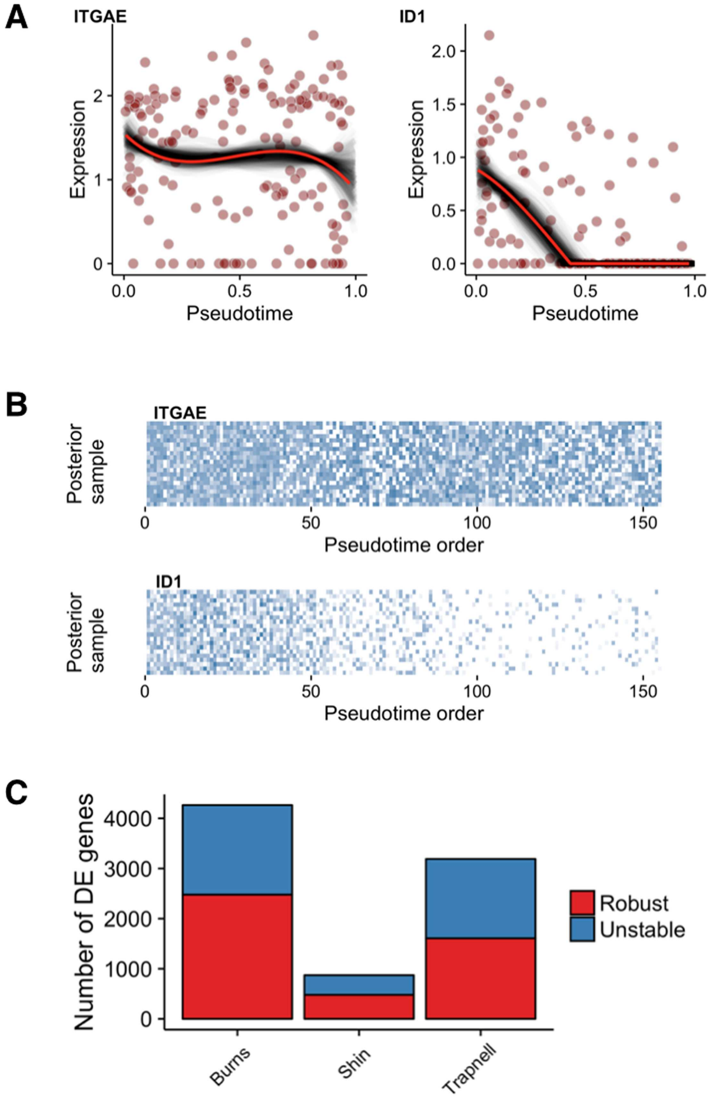

Fig 6. Approximate FDR for differential expression across pseudotime. (A) Gene expression plots across pseudotime, with black traces corresponding to models fitted to pseudotime samples while the red trace corresponds to the point (MAP) estimate for two exemplar genes and **(B)** corresponding gene expression heatmap for 20 randomly sampled posterior pseudotimes. **(C)** The number of genes identified as robust and unstable for all three datasets examined.

doi:10.1371/journal.pcbi.1005212.g006

ID1—known to be involved in muscle differentiation—had a q-value of 6.6×10^{-11} using the MAP pseudotime estimate, but was also called significant in all the posterior pseudotime estimates having a median q-value of 4.4×10^{-11}. This indicates that the significance of the temporal expression variability of ID1 is highly robust whilst the significance ITGAE is much more dependent on the exact pseudotemporal ordering chosen.

As a conservative rule of thumb, we designated two sets of putative temporal associations: (i) a *robust* set comprising genes with a q-value less than 5% at the MAP estimate of pseudotime but also identified as significant in 95+% of the posterior pseudotime samples and (ii) an *unstable* set of genes whose q-values are less than 5% using the MAP estimate of pseudotime

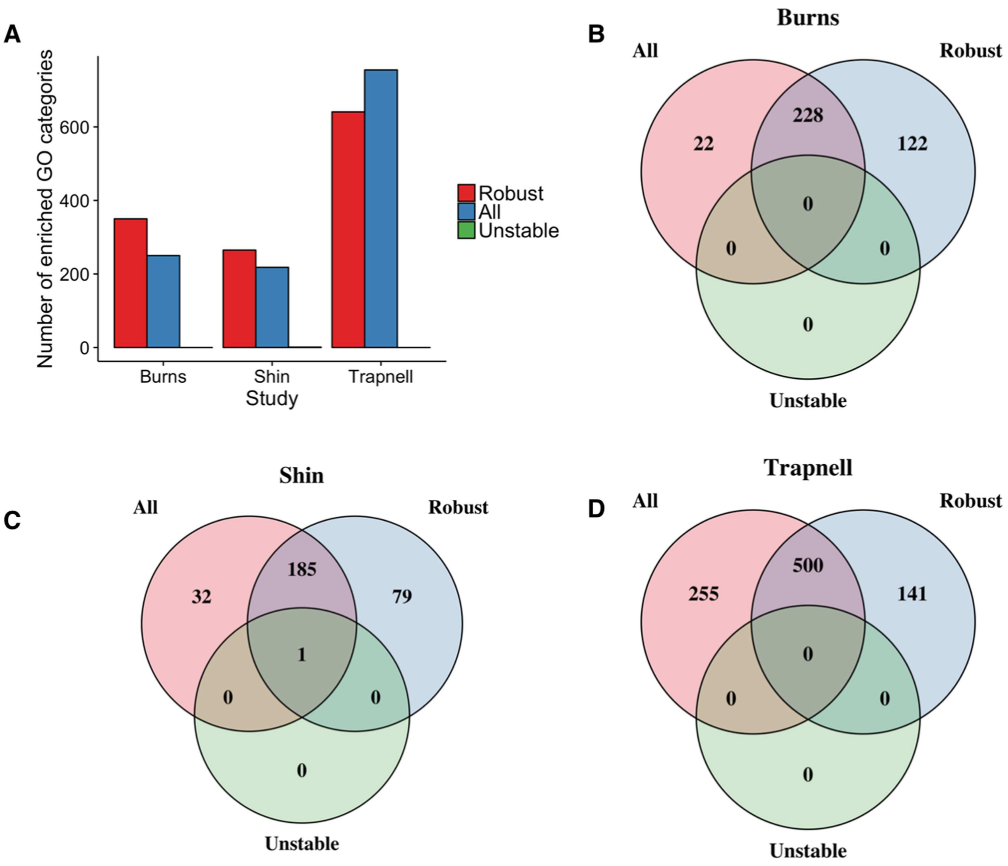

Fig 7. Gene Ontology Enrichment Analysis. (A) Number of enriched GO categories for the three datasets studied. Genes used for enrichment were either those that exhibit *robust* differential expression, *unstable* differential expression or *all*. **(B-D)** Venn diagrams showing the number of enriched GO terms based on the differential expression categories above.

doi:10.1371/journal.pcbi.1005212.g007

but is significant in less than 95% of the posterior pseudotime samples. Looking across all genes in the the three datasets we found that approximately half of the associations in all three datasets were unstable and whose temporal association depended on the choice of pseudotime estimates (Fig 6C).

We performed a Gene Ontology enrichment analysis of the differentially expressed genes using (i) the robust set only, (ii) all DE genes (robust and unstable) and (iii) the unstable set only, using the GOseq package [36] with a 5% FDR significance level, enriching for categories corresponding to biological processes (Fig 7). The set of unstable genes give no significant enriched GO categories on their own whilst the robust set gave a similar number of enriched GO categories as using all DE genes despite containing only half the number of genes. In all three datasets, there was a large overlap between the enriched GO categories identified and interestingly a high proportion of GO terms that are only significant from the robust gene set only. This suggests that the inclusion of the unstable gene set, potentially containing nuisance findings, may have reduced power to identify certain GO categories. Overall, our analysis suggests that the robust set of temporally differentially expressed genes identified by taking into account posterior uncertainty is a biologically meaningful gene set and not an arbitrary subset of the set of all DE genes.

A sigmoidal model of switch-like behaviour across pseudotime

In the previous section, we examined differential expression across pseudotime by fitting generalized additive models to the gene expression profiles [12]. Their approach used a Tobit regression model with a cubic smoothing spline link function. Hypothesis testing using the likelihood ratio test is conducted against a null model of no pseudotime dependence. This model provides a highly flexible but non-specific model of pseudotime dependence that was not suited to the next question we wished to address.

Specifically, we were interested in whether we could identify if two genes switched behaviours at the *same* (or similar) times during the temporal process and therefore an estimate of the time resolution that can be gained from a pseudotime estimation approach. This requires estimation of a parameter that can be directly linked to a switch on(/off) time that is not present in the Tobit regression model. As a result, we propose a "sigmoidal" model of differential expression across pseudotime that better captures switch-like gene (in)activation and has easy to interpret parameters corresponding to activation time and strength. By combining such a parametric model with the Bayesian inference of pseudotime we can then infer the resolution to which we can say whether one gene switches on or off before another. Details of the sigmoidal gene activation model are given in Methods and in S1 Text.

We applied our sigmoidal model to learn patterns of switch-like behaviour of genes in the Trapnell dataset. For each gene we estimated the *activation time* (t_0) as well as the *activation strength* (k). We fitted these sigmoidal switching models to all posterior pseudotime samples to approximate the posterior distribution for the time and strength parameters. We uncovered a small set of genes whose median activation strength is distinctly larger than the rest and had low variability across posterior pseudotime samples implying a population of genes that exhibit highly switch-like behaviour (Fig 8A). Some genes showed high activation strength for certain pseudotime estimates but low overall median levels across all the posterior samples. We concluded that genes with large credible intervals on the estimates of activation strength do not show robust switch-like behaviour and demonstrate the necessity of using probabilistic methods to infer gene behaviour as opposed to point estimates that might give highly unstable results.

Representative examples of genes with large and small activation strengths showed marked differences in the gene expression patterns corresponding to strong and weak switch-like behaviour as expected (Fig 8B). In addition, we examined the posterior density activation time t_0 for the five genes showing strong switching behaviour (Fig 8C). Under a point estimate of pseudotime each gene would give a distinct activation time with which these genes can be ordered. However, when pseudotime uncertainty is taken into account, a distribution over possible activation times emerges. In this case, the five genes all have activation times between 0.3 and 0.5 precluding a precise ordering (if one exists) of activation. Visually, this seems sensible since there is considerable cell-to-cell variability in the expression of these genes and not all cells express the genes during the "on" phase. We are therefore unable to determine whether the "on" phase begins when the first cell with high expression is first observed in pseudotime or, if it starts before, and the first few cells simply have null expression (for biological or technical reasons).

We further explore this in Fig 9 which shows ten genes identified as having significant switch-like pseudotime dependence but with a range of mean activation times t_0. The switch-like behaviour is stable to the different posterior pseudotime estimates that were sampled from the GPLVM. It is clear that the two genes RARRES3 and C1S are activating at an earlier time compared to the genes IL20RA and APOL4. However, we cannot be confident of the ordering within the pairs RARRES3/C1S and IL20RA/APOL4 in pseudotime since the distributions

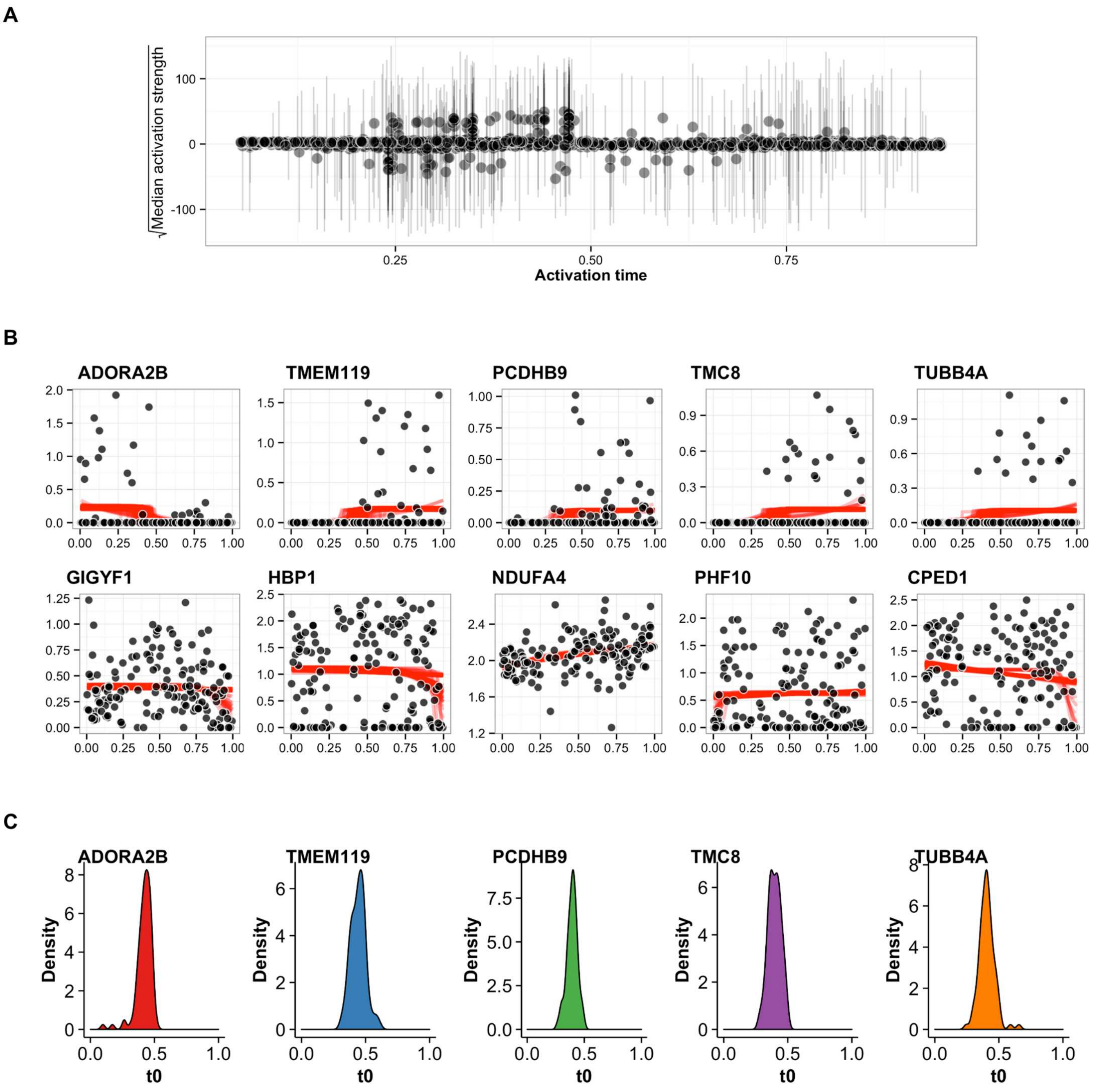

Fig 8. Robust inference of switch-like behaviour in genes across pseudotime. (A) The square-root of the median of the activation strength parameter *k* across all pseudotime samples as a function of activation time t_0. The error bars show the 95% credible interval, demonstrating that point estimates can severely skew the apparent behaviour of genes and a requirement for a robust Bayesian treatment of gene expression. A distinct population of genes whose median activation strength sits separate from the majority close to the x-axis implies a subset of genes show true switch-like behaviour. **(B)** Representative examples of genes whose median activation strength is large (top row) compared to small (bottom row). Each black point represents the gene expression of the cell with red lines corresponding to posterior traces of the sigmoidal gene expression model. Genes with a large activation strength show a distinct gene expression pattern compared to those with a small activation strength. **(C)** A posterior density plot of the activation time for the five genes showing strong activation strength in (B).

doi:10.1371/journal.pcbi.1005212.g008

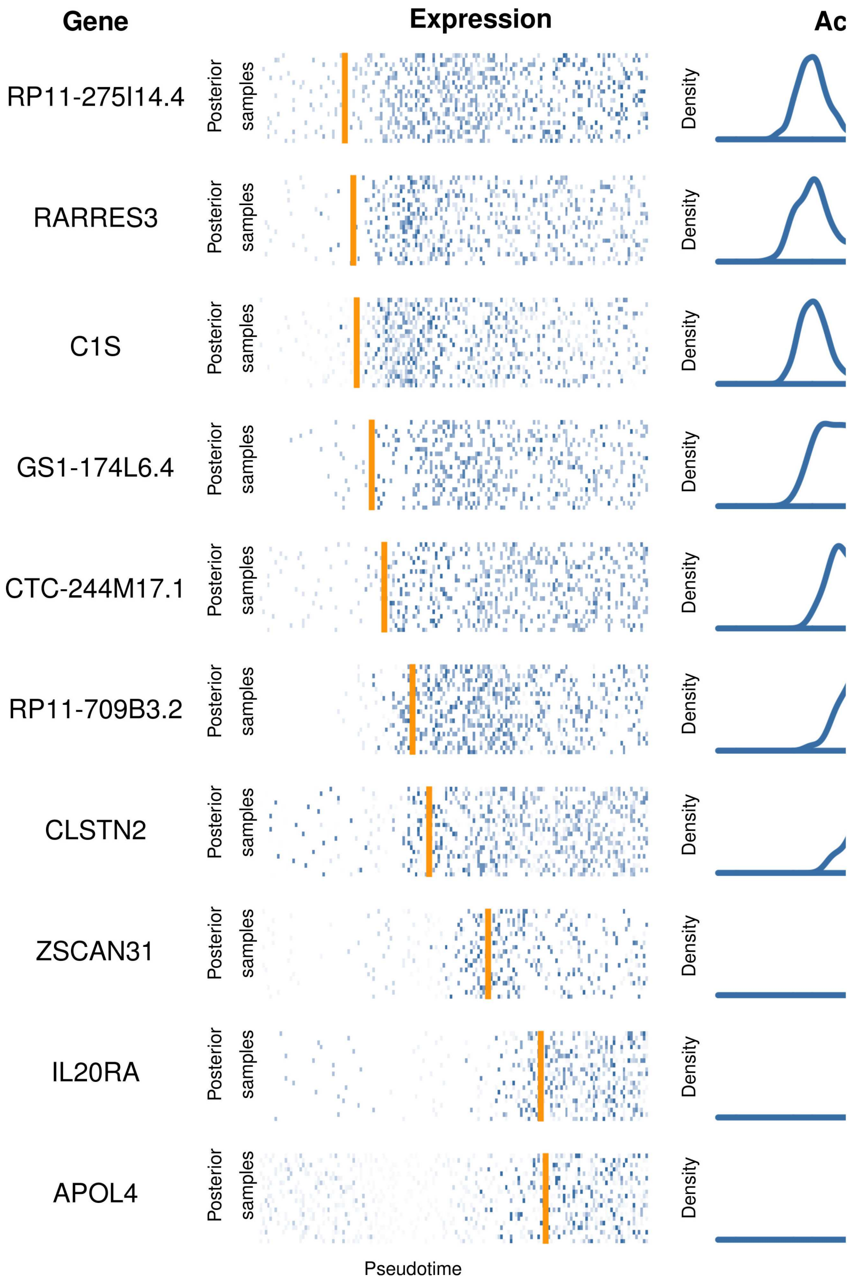

Fig 9. Identifying pseudotime dependent gene activation behaviour. Ten selected genes from [12] found using our sigmoidal gene activation model exhibiting a range of activation times. For each gene, we show the expression levels of each cell (centre) where each row corresponds to an ordering according to a different posterior samples of pseudotime. The orange line corresponds to a point estimate of the activation time. The posterior density of the estimated activation time is also shown (right).

doi:10.1371/journal.pcbi.1005212.g009

over the activation times are not well-separated and it is impossible to make any definitive statements as to whether one of these genes (in)activates before another. If the probability of a sequence of activation events is required, instead of examining each gene in isolation, we can count the number of posterior samples in which one gene precedes another instead and evidence may emerge of a possible ordering. These observations suggests a finite temporal resolution limit that can be obtained using pseudotemporal ordering.

We note that we have deliberately avoid directly linking the sigmoidal gene activation and GPLVM pseudotime models to derive a single, joint model. In a joint model, the inference would attempt to order the cells in such a way as to maximise the fit of the sigmoidal and GPLVM to the expression data. However, as the sigmoidal model is only intended to identify genes with switch-like behaviour, it cannot explain other types of pseudotime dependence that may and do exist. This model misspecification would potentially drive inference in ways that cannot be foreseen.

Contribution to pseudotime uncertainty from the reduced dimensional representation

Finally, we address the impact of the dimensionality reduction that is often applied to single cell gene expression data prior to pseudotime estimation. The choice of dimensionality reduction approach is based on whether the method gives rise to a putative pseudotime trajectory in the reduced dimensionality representation. This is typically conducted with visual inspection followed by confirmational analysis by examining known marker genes with established temporal association. This may lead to a number of possibilities since the same trajectory may exist in a number of reduced dimensionality representations.

We sought to characterise the contribution of the dimensionality reduction process to pseudotime uncertainty. The wide variety of dimensionality reduction methods available and in use for pseudotime estimation precludes a complete investigation here and we choose to focus instead on principal components analysis—a standard technique—and used, for example, in Waterfall [37] or TSCAN [18] or as a preprocessing step used before applying non-linear methods such as t-SNE or GPLVMs.

To do this, we used the differentiating myoblasts data set [12] and performed PCA (using the `R` package `scater` [38]). We then applied GPLVM pseudotime estimation to the two-dimensional principal component representation and identified a set of 1,968 robustly differentially expressed genes from the posterior pseudotime samples. Next, we took 50 random subsets containing 80% of the cells and repeated the above procedure on each subset. As the PCA projection depends on the data itself, the reduced dimensional representation derived from each subsample will be different, we wanted to compare the differentially expressed genes identified to those found using the full data to determine if the variation in the reduced dimensionality representation impacts on the downstream differential expression analyses. We examined all genes and PCA subsamples and found that over 90% of differential expression tests showed a robust temporal association (Fig 10A). Over half of all genes were robustly differentially expressed in all PCA subsamples and over 80% in at least 40 out of 50 PCA subsamples (Fig 10B). These results indicate that the vast majority of genes remained robustly differentially expressed despite the differences in the reduced dimensional space across the random subsets (and the potential loss of detection power due to there being fewer cells).

Our results may be further explained by the fact that principal components analysis uses a linear, orthogonal transformation that maximises variance in the principal directions. This will give reduced dimensional representations that are likely to be robust in many instances to variable cell inputs given sufficiently large sample sizes. Highly nonlinear techniques, such as t-SNE, Laplacian Eigenmaps or diffusion maps, maybe more sensitive to the input data and the resultant reduced dimensional representations more variable. A thorough characterisation of the relative contribution of the reduced dimensional representation and the curve fitting to the statistical uncertainty in the pseudotime estimates must be determined through simulations (like the ones detailed here) and conclusions may differ for different dimensionality reduction/curve fitting combinations.

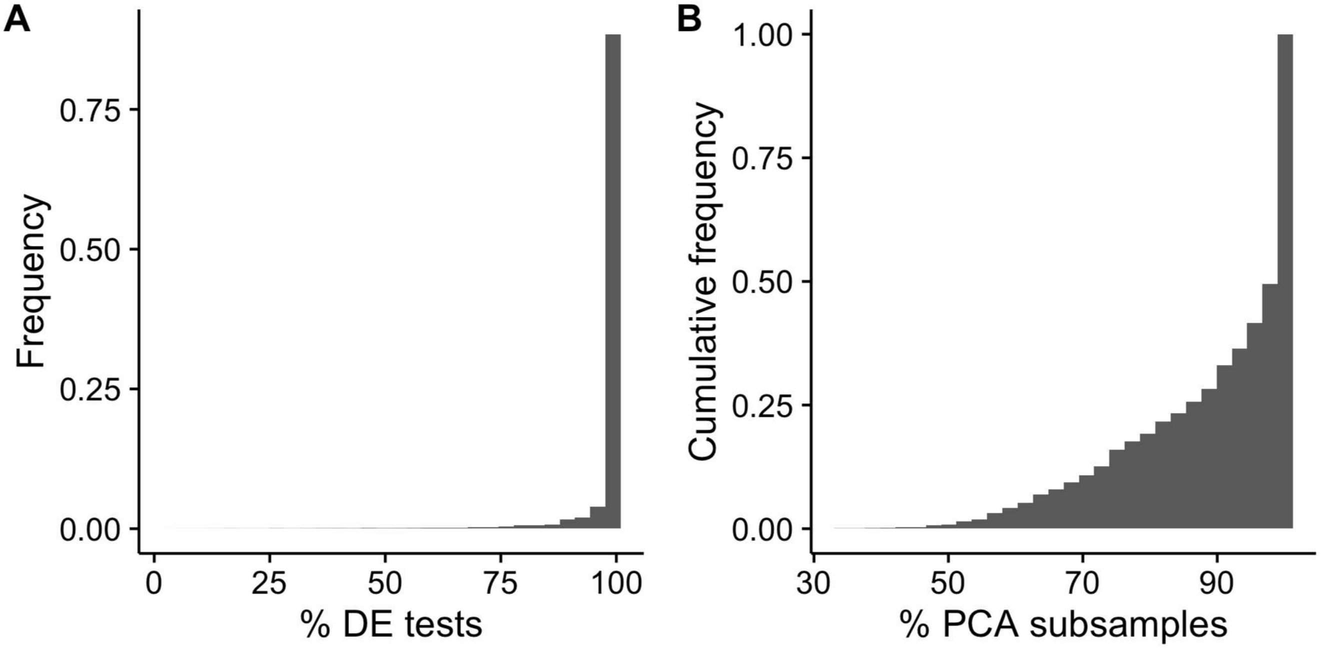

Fig 10. Pseudotime uncertainty arising from reduced dimensional representation. (A) Proportion of all subsampled differential expression tests where the gene was found to be robustly differentially expressed using only those genes robustly differentially expressed when considering all cells. **(B)** Cumulative frequency of cell subsamples in which a given gene is robustly differentially expressed. Over half of all genes were robustly differentially expressed in all PCA subsamples.

doi:10.1371/journal.pcbi.1005212.g010

Discussion

Pseudotime estimation from gene expression profiling of single cells provides the ability to extract temporal information about complex biological processes from which *true* time series experimentation may be technically challenging or impossible. In our investigations we have sought to characterise the utility of a probabilistic approach to the single cell pseudotime estimation problem over approaches that only return a single point estimate of pseudotime. Our work is significant since it has so far not been possible to assess the impact of this statistical uncertainty in downstream analyses and to ascertain the level of temporal resolution that can be obtained.

In order to address this we adopted a Gaussian Process Latent Variable modelling framework to perform probabilistic pseudotime estimation within a Bayesian inference setting. The GPLVM allows us to probabilistically explore a range of different pseudotime trajectories within the reduced dimensional space. We showed that in a truly unbiased and unsupervised analysis the properties of the pseudotime trajectory will never purely be a product of the data alone and can heavily depend on prior assumptions about the smoothness, length scales of the trajectory and noise properties. Using samples drawn from the posterior distribution over pseudotime estimates under the GPLVM we were able to assess if genes that showed a significant pseudotime dependence under a point (MAP) pseudotime estimate would be robust to different possible pseudotime estimates. In two of the three datasets we examined we discovered that, when adjusted for pseudotime uncertainty, the false discovery rate may be significantly larger than the target 5%. Our investigations show that reliance on a single estimate of pseudotime can lead to increased number of false discoveries but that it is possible to assess the impact of such assumptions within a probabilistic framework.

A caveat of the specific methodology adopted in this study is that it is necessarily computationally intensive due to the use of full Markov chain Monte Carlo based Bayesian inference and is dominated by functions of the Gaussian Process covariance matrix that have complexity $O(n^3)$ where n is the number of cells. Our STAN implementation is able to process 300 cells in around 15 minutes on a standard laptop computer but well-known variational approximations based on inducing point [39] and recent stochastic variational algorithms [40] can reduce the computational burden and improve scaling to $O(n) > 10^4$. However, this scalability comes at the expense of the reduced ability to fully characterise posterior uncertainty. In this study we have focused purely on best characterising the posterior uncertainty using MCMC algorithms that asymptotically converge to the true posterior distribution. In practice this purest approach may not be necessary but we argue that pseudotime uncertainty should be addressed.

It is important to note that the GPLVM used in our investigations is not intended to be a single, all-encompassing solution for pseudotime modelling problems. For our purposes, it provided a simple and relevant device for tackling the single trajectory pseudotime problem in a probabilistic manner but clearly has limitations when the temporal process under investigation contains bifurcations or heteroscedastic noise processes (as discussed earlier). Improved and/or alternative probabilistic models are required to address more challenging modelling scenarios but the general procedures we describe are generic and should be applicable to any problem where statistical inference for a probabilistic model can give posterior simulation samples.

We also developed a novel sigmoidal gene expression temporal association model that enabled us to identify genes exhibiting a strong switch-like (in)activation behaviour. For these genes we were then able to estimate the activation times and use these to assess the time resolution that can be attained using pseudotime estimates of single cells. Our investigations show that pseudotime uncertainty prevents precise characterisation of the gene activation time but a probabilistic model can provide a distribution over the possibilities. In application, this uncertainty means that it is challenging to make precise statements about when regulatory factors will turn on or off and if they act in unison. This places an upper limit on the accuracy of dynamic gene regulation models and causal relationships between genes that could be built from the single cell expression data.

In conclusion, single cell genomics has provided a precision tool with which to interrogate complex temporal biological processes. However, as widely reported in recent studies, the properties of single cell gene expression data are complex and highly variable. We have shown that the many sources of variability can contribute to significant uncertainty in statistical inference for pseudotemporal ordering problems. We argue therefore that strong statistical foundations are vital and that probabilistic methods for provide a platform for quantifying uncertainty in pseudotemporal ordering which can be used to more robustly identify genes that are differentially expressed over time. Robust statistical procedures can also temper potentially unrealistic expectations about the level of temporal resolution that can be obtained from computationally-based pseudotime estimation. Ultimately, as the raw input data is not true time series data, pseudotime estimation is only ever an attempt to solve a *missing data* statistical inference problem that we should remind ourselves involves quantities (pseudotimes) that are *unknown, never can be known.*

Supporting Information

S1 Text. Supplementary Methods.
(PDF)

S1 Fig. Inherent uncertainty from sub-sampling with point-estimation methods. 80% of cells were subsampled from the Laplacian Eigenmaps representation 30 times and the pseudotime refitted using `Monocle`'s MST approach. **A** Boxplots of resamples across all cells. Upper and lower whiskers extend to the highest and lowest values within 1.5 times the interquartile range. **B** The 2σ interval was then computed for the pseudotime of each cell, which varies from as low as close to 0 up to almost half the pseudotime interval (0.5).
(TIF)

S2 Fig. Stability of trajectories across multiple chains. GPLVM trajectories were fit on the Trapnell dataset using either a high level of shrinkage in **A** & **C** ($\gamma_\alpha = 35$, $\gamma_\beta = 5$) or using a low level of shrinkage in **B** & **D** ($\gamma_\alpha = 3$, $\gamma_\beta = 1$) for 10 separate MCMC chains. It can be seen that the trajectories consistently fit using high levels of shrinkage, implying this is required to have a well-defined posterior as opposed to a 'lumpy' posterior with many local maxima using low levels of shrinkage. **C** & **D** show the posterior densities for a randomly chosen cell (number 100) using the two different shrinkage regimes as defined above.
(TIF)

S3 Fig. Comparison of MAP pseudotime using GPLVM to orderings using `Monocle`. Left: `Monocle` ordering found using ICA on 500 most variable genes. Right: `Monocle` orderings found using a Laplacian Eigenmaps embedding as described above. Error bars show the 95% HPD credible interval.
(TIF)

Author Contributions

Conceived and designed the experiments: KRC CY.

Performed the experiments: KRC.

Analyzed the data: KRC.

Contributed reagents/materials/analysis tools: KRC.

Wrote the paper: KRC CY.

References

1. Kalisky T, Quake SR. Single-cell genomics. Nature methods. 2011; 8(4):311–314. doi: 10.1038/nmeth0411-311 PMID: 21451520
2. Shapiro E, Biezuner T, Linnarsson S. Single-cell sequencing-based technologies will revolutionize whole-organism science. Nature reviews Genetics. 2013 Sep; 14(9):618–30. Available from: http://www.ncbi.nlm.nih.gov/pubmed/23897237. doi: 10.1038/nrg3542 PMID: 23897237
3. Macaulay IC, Voet T. Single cell genomics: advances and future perspectives. PLoS genetics. 2014 Jan; 10(1):e1004126. Available from: http://www.pubmedcentral.nih.gov/articlerender.fcgi?artid=3907301&tool=pmcentrez&rendertype=abstract. doi: 10.1371/journal.pgen.1004126 PMID: 24497842
4. Wills QF, Mead AJ. Application of Single Cell Genomics in Cancer: Promise and Challenges. Human molecular genetics. 2015;p. ddv235. doi: 10.1093/hmg/ddv235 PMID: 26113645
5. Stegle O, Teichmann Sa, Marioni JC. Computational and analytical challenges in single-cell transcriptomics. Nature reviews Genetics. 2015 Jan; 16(3):133–145. Available from: http://www.nature.com/doifinder/10.1038/nrg3833. PMID: 25628217
6. Trapnell C. Defining cell types and states with single-cell genomics. Genome Res. 2015 Oct; 25(10):1491–8. doi: 10.1101/gr.190595.115 PMID: 26430159
7. Treutlein B, Brownfield DG, Wu AR, Neff NF, Mantalas GL, Espinoza FH, et al. Reconstructing lineage hierarchies of the distal lung epithelium using single-cell RNA-seq. Nature. 2014; 509(7500):371–375. doi: 10.1038/nature13173 PMID: 24739965

8. Tsang JC, Yu Y, Burke S, Buettner F, Wang C, Kolodziejczyk AA, et al. Single-cell transcriptomic reconstruction reveals cell cycle and multi-lineage differentiation defects in Bcl11a-deficient hematopoietic stem cells. Genome biology. 2015; 16(1):1–16. doi: 10.1186/s13059-015-0739-5 PMID: 26387834

9. Qiu P, Simonds EF, Bendall SC, Gibbs KD Jr, Bruggner RV, Linderman MD, et al. Extracting a cellular hierarchy from high-dimensional cytometry data with SPADE. Nature biotechnology. 2011; 29(10):886–891. doi: 10.1038/nbt.1991 PMID: 21964415

10. Bendall SC, Davis KL, Amir EAD, Tadmor MD, Simonds EF, Chen TJ, et al. Single-cell trajectory detection uncovers progression and regulatory coordination in human B cell development. Cell. 2014 Apr; 157(3):714–25. Available from: http://www.sciencedirect.com/science/article/pii/S0092867414004711. doi: 10.1016/j.cell.2014.04.005 PMID: 24766814

11. Marco E, Karp RL, Guo G, Robson P, Hart AH, Trippa L, et al. Bifurcation analysis of single-cell gene expression data reveals epigenetic landscape. Proceedings of the National Academy of Sciences of the United States of America. 2014 Dec; 111(52):E5643–50. Available from: http://www.pubmedcentral.nih.gov/articlerender.fcgi?artid=4284553&tool=pmcentrez&rendertype=abstract. doi: 10.1073/pnas.1408993111 PMID: 25512504

12. Trapnell C, Cacchiarelli D, Grimsby J, Pokharel P, Li S, Morse M, et al. The dynamics and regulators of cell fate decisions are revealed by pseudotemporal ordering of single cells. Nature biotechnology. 2014 Apr; 32(4):381–6. Available from: http://www.ncbi.nlm.nih.gov/pubmed/24658644. doi: 10.1038/nbt.2859 PMID: 24658644

13. Moignard V, Woodhouse S, Haghverdi L, Lilly AJ, Tanaka Y, Wilkinson AC, et al. Decoding the regulatory network of early blood development from single-cell gene expression measurements. Nature Biotechnology. 2015 Feb; 33(3). Available from: http://www.nature.com/doifinder/10.1038/nbt.3154. PMID: 25664528

14. Reid JE, Wernisch L. Pseudotime estimation: deconfounding single cell time series. bioRxiv. 2015;p. 019588. doi: 10.1093/bioinformatics/btw372 PMID: 27318198

15. Hanchate NK, Kondoh K, Lu Z, Kuang D, Ye X, Qiu X, et al. Single-cell transcriptomics reveals receptor transformations during olfactory neurogenesis. Science. 2015 Nov;p. science.aad2456–. Available from: http://www.sciencemag.org/content/early/2015/11/04/science.aad2456.full. doi: 10.1126/science.aad2456 PMID: 26541607

16. Shin J, Berg DA, Zhu Y, Shin JY, Song J, Bonaguidi MA, et al. Single-Cell RNA-Seq with Waterfall Reveals Molecular Cascades underlying Adult Neurogenesis. Cell Stem Cell. 2015 Aug; 17(3):360–372. Available from: http://www.cell.com/article/S1934590915003124/fulltext. doi: 10.1016/j.stem.2015.07.013 PMID: 26299571

17. Haghverdi L, Buettner M, Wolf FA, Buettner F, Theis FJ. Diffusion pseudotime robustly reconstructs lineage branching. Nature Methods. 2016;. doi: 10.1038/nmeth.3971 PMID: 27571553

18. Ji Z, Ji H. TSCAN: Pseudo-time reconstruction and evaluation in single-cell RNA-seq analysis. Nucleic acids research. 2016;p. gkw430. doi: 10.1093/nar/gkw430 PMID: 27179027

19. Matsumoto H, Kiryu H. SCOUP: a probabilistic model based on the Ornstein–Uhlenbeck process to analyze single-cell expression data during differentiation. BMC bioinformatics. 2016; 17(1):1. doi: 10.1186/s12859-016-1109-3 PMID: 27277014

20. Magwene PM, Lizardi P, Kim J. Reconstructing the temporal ordering of biological samples using microarray data. Bioinformatics. 2003; 19(7):842–850. doi: 10.1093/bioinformatics/btg081 PMID: 12724294

21. Gupta A, Bar-Joseph Z. Extracting dynamics from static cancer expression data. IEEE/ACM transactions on computational biology and bioinformatics / IEEE, ACM. 2008; 5(2):172–82. Available from: http://www.ncbi.nlm.nih.gov/pubmed/18451427. doi: 10.1109/TCBB.2007.70233 PMID: 18451427

22. Qiu P, Gentles AJ, Plevritis SK. Discovering biological progression underlying microarray samples. PLoS computational biology. 2011 Apr; 7(4):e1001123. Available from: http://www.pubmedcentral.nih.gov/articlerender.fcgi?artid=3077357&tool=pmcentrez&rendertype=abstract. doi: 10.1371/journal.pcbi.1001123 PMID: 21533210

23. Haghverdi L, Buettner F, Theis FJ. Diffusion maps for high-dimensional single-cell analysis of differentiation data. Bioinformatics. 2015;(May:):1–10. Available from: http://www.ncbi.nlm.nih.gov/pubmed/26002886. doi: 10.1093/bioinformatics/btv325 PMID: 26002886

24. Hinton GE, Roweis ST. Stochastic neighbor embedding. In: Advances in neural information processing systems; 2002. p. 833–840.

25. Van der Maaten L, Hinton G. Visualizing data using t-SNE. Journal of Machine Learning Research. 2008; 9(2579–2605):85.

26. Amir EaD, Davis KL, Tadmor MD, Simonds EF, Levine JH, Bendall SC, et al. viSNE enables visualization of high dimensional single-cell data and reveals phenotypic heterogeneity of leukemia. Nature biotechnology. 2013 Jun; 31(6):545–52. Available from: http://www.ncbi.nlm.nih.gov/pubmed/23685480. doi: 10.1038/nbt.2594 PMID: 23685480

27. Hastie T, Stuetzle W. Principal Curves. 2012 Mar;Available from: http://www.tandfonline.com/doi/abs/10.1080/01621459.1989.10478797.

28. Campbell K, Yau C. Bayesian Gaussian Process Latent Variable Models for pseudotime inference in single-cell RNA-seq data. bioRxiv. 2015;p. 026872.

29. Macaulay IC, Svensson V, Labalette C, Ferreira L, Hamey F, Voet T, et al. Single-Cell RNA-Sequencing Reveals a Continuous Spectrum of Differentiation in Hematopoietic Cells. Cell Reports. 2016;. doi: 10.1016/j.celrep.2015.12.082 PMID: 26804912

30. Gelman A, Lee D, Guo J. Stan A Probabilistic Programming Language for Bayesian Inference and Optimization. Journal of Educational and Behavioral Statistics. 2015;p. 1076998615606113. doi: 10.3102/1076998615606113

31. Pierson E, Yau C. ZIFA: Dimensionality reduction for zero-inflated single-cell gene expression analysis. Genome biology. 2015; 16(1):1–10. doi: 10.1186/s13059-015-0805-z PMID: 26527291

32. Titsias M, Lawrence N. Bayesian Gaussian Process Latent Variable Model. Artificial Intelligence. 2010; 9:844–851. Available from: http://eprints.pascal-network.org/archive/00006343/.

33. Burns JC, Kelly MC, Hoa M, Morell RJ, Kelley MW. Single-cell RNA-Seq resolves cellular complexity in sensory organs from the neonatal inner ear. Nature Communications. 2015; 6:8557. Available from: http://www.nature.com/doifinder/10.1038/ncomms9557. PMID: 26469390

34. Belkin M, Niyogi P. Laplacian Eigenmaps for Dimensionality Reduction and Data. 2003; 1396:1373–1396.

35. Le QV, Smola AJ, Canu S. Heteroscedastic Gaussian process regression. In: Proceedings of the 22nd international conference on Machine learning. ACM; 2005. p. 489–496.

36. Young MD, Wakefield MJ, Smyth GK, Oshlack A. Gene ontology analysis for RNA-seq: accounting for selection bias. Genome biology. 2010; 11(2):1. doi: 10.1186/gb-2010-11-2-r14 PMID: 20132535

37. Shin J, Berg DA, Zhu Y, Shin JY, Song J, Bonaguidi MA, et al. Single-cell RNA-seq with waterfall reveals molecular cascades underlying adult neurogenesis. Cell stem cell. 2015; 17(3):360–372. doi: 10.1016/j.stem.2015.07.013 PMID: 26299571

38. McCarthy D, Wills Q, Campbell K. scater: Single-cell analysis toolkit for gene expression data in R.;. Https://github.com/davismcc/scater.

39. Titsias MK. Variational Learning of Inducing Variables in Sparse Gaussian Processes. In: AISTATS. vol. 12; 2009. p. 567–574.

40. Hensman J, Fusi N, Lawrence ND. Gaussian processes for big data. arXiv preprint arXiv:13096835. 2013;.
